# Tibialis anterior and extensor hallucis longus tendon ruptures complicating a closed tibial fracture: A case report

**DOI:** 10.1016/j.ijscr.2024.110388

**Published:** 2024-09-30

**Authors:** Saber Barazandeh Rad, Mehrdad Sadighi, Meisam Jafari Kafiabadi, Adel Ebrahimpour, Mohammad Pourmahmoudian, Amin Karami

**Affiliations:** Department of Orthopedic Surgery, Clinical Research Development Unit of Shohada-e Tajrish Hospital, Shahid Beheshti University of Medical Sciences, Tehran, Iran

**Keywords:** Tibial fracture, Tibialis anterior tendon, Extensor hallucis longus tendon, Tendon rupture, Tendon repair

## Abstract

**Introduction and importance:**

Acute rupture of the tibialis anterior tendon and other tendons is an extremely rare injury. It is usually associated with minor trauma in older patients with medical comorbidity. Surgeons must be alert for rupture of these tendons which can complicate a closed tibial fracture.

**Case presentation:**

A 19-year-old man was transferred to the emergency department with a segmental fracture at the lower third of his tibia. The patient could not actively dorsiflex his right ankle and first toe but was able to dorsiflex other toes actively. The tibialis anterior and extensor hallucis longus tendon were discovered torn at the same point, supposedly by a bone spike. The fracture was reduced and fixed and the tendons were repaired.

**Clinical discussion:**

A traumatic rupture of the tibialis anterior and extensor hallucis longus tendons occurs rarely in conjunction with or as a consequence of a bony fracture in closed trauma, with only a few cases documented in the literature. They typically occur as a result of direct blunt or penetrating injury. In three papers, the tibial fracture caused a direct rupture in the tendon. We believe that the tendons rubbing against the fractured edges of the segmental bone of the tibia led to the tendons tearing gradually.

**Conclusion:**

Further investigation is needed for an evaluation of ankle and first toe dorsiflexion to check for a palpable gap in the soft tissues of a lower third tibial fracture. The tibialis anterior and other tendons can be located between the segment of the tibial fracture and ruptured.

## Highlights

• Rupture of the tibialis anterior tendon complicating a closed tibial fracture is rare.
• Palpable gap in the soft tissues of the anterior aspect of the lower third tibia fracture requires dorsiflexion examination.
• Careful examination of the tibialis anterior tendon should always be done in cases of oblique tibial fractures.

## 1 Introduction

Acute ruptures of the tibialis anterior and extensor hallucis longus tendon are very rare injury. It is usually associated with minor trauma and occurs in patients older than 45 years of age who are suffering from diabetes mellitus, hypothyroidism, gout, martial arts, arthritic adhesion, psoriasis or after a local steroid injection [[1], [2], [3], [4], [5], [6], [7], [8], [9], [10]]. However, to our knowledge, with only a handful of cases described in the literature, the pathoanatomy and best imaging method are not well described and illustrated of acute traumatic laceration of the tibialis anterior tendon after a closed tibial fracture [[11], [12], [13], [14], [15], [16], [17]]. The traumatic laceration of the tibialis anterior tendon was suspected only on follow-up examination, 2 weeks to 11 months after injury, when the patient's inability to actively dorsiflex the ankle joint became apparent.

Early repair of tibialis anterior and extensor hallucis longus tendon ruptures, especially in active young adults, is essential and leads to excellent results, as opposed to late or no repair, which may cause a severe functional deficit [[2], [3], [4],[8], [9], [10],^12^]. Markarian et al. showed that nonsurgical treatment of such cases is only acceptable in ruptures occurring in elderly low-demand patients [17].

This case report aims to present a case to highlight the pitfalls that must alert the surgeon at acute traumatic laceration of the tibialis anterior and extensor hallucis longus tendon complicating a closed tibial fracture in a pedestrian car accident in recognition as well as in treatment of this rare condition.

## 2 Case presentation

A 19-year-old man was transferred to the accident and emergency department after a motorbike car accident. He slipped and fell sideways, sustaining a twisting injury to his left leg as it became entrapped between his motorbike and the ground. The leg was swollen and painful unable to weight bearing. No neurovascular damage was noted and there were no clinical signs of a compartment syndrome present. The patient was unable to actively dorsiflex his right ankle and first toe, whereas active dorsiflexion of other toes could be performed.

**Fig. 1:**
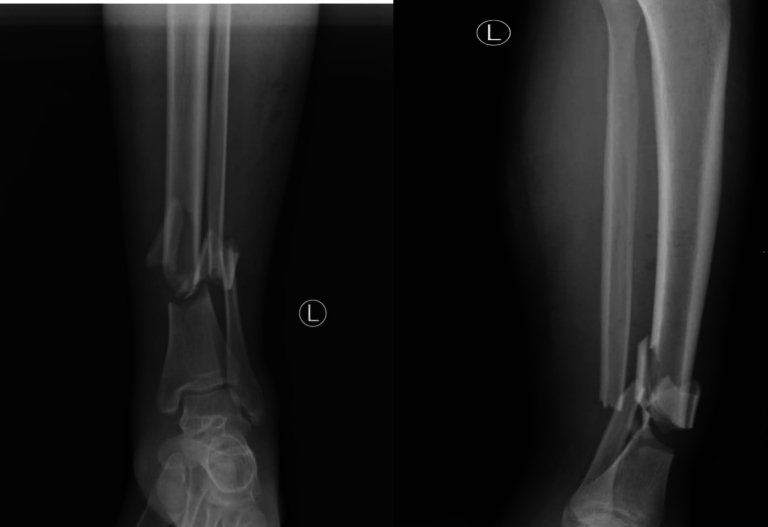
Preoperative anteroposterior and lateral radiographic imaging showing segmental fracture at the junction of the middle and distal third of his tibia.

A careful physical examination determined a palpable gap in the soft tissues over the anterolateral aspect of the lower third of his right tibia. Plain radiographic imaging showed a segmental fracture at the junction of the middle and distal third of his tibia (Fig. 1). The palpable gap in the soft tissues and his inability to actively dorsiflex his right ankle and first toe led us to strongly suspect that the tibialis anterior and extensor hallucis longus tendons were acutely injured from the bony spike or were entrapped within the fracture site.

**Fig. 2:**
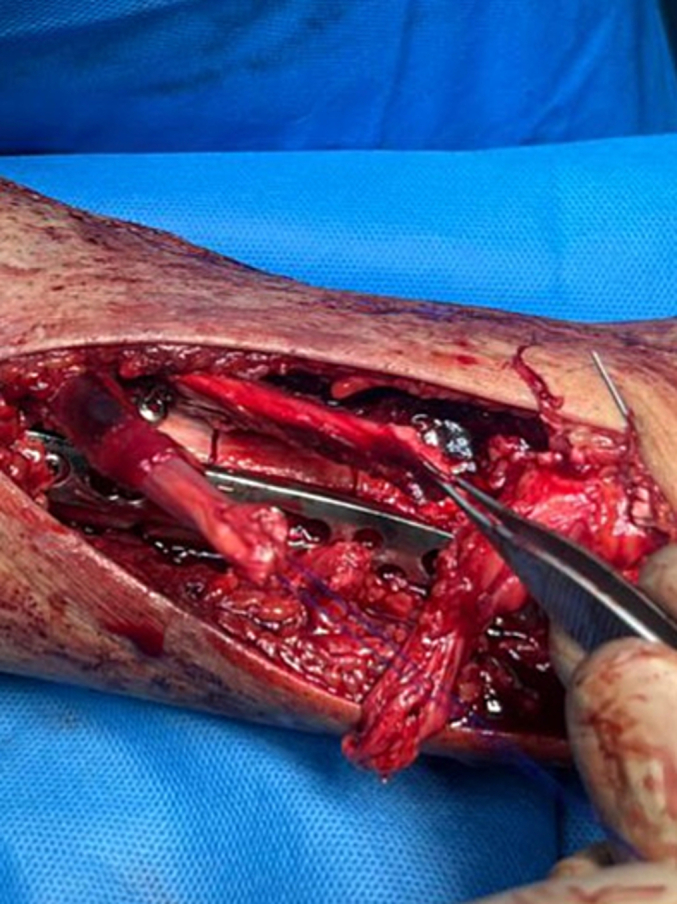
Tibialis anterior and extensor hallucis longus tendon tear at the same level of fracture site with its stumps.

Open reduction and internal fixation of the fracture were chosen to simultaneously explore the tibialis anterior and extensor hallucis longus tendons. The fracture site was exposed via an incision over the anteromedial aspect of the distal half of the tibia. The tibialis anterior and extensor hallucis longus tendons were found lacerated at the same level with their stumps cleanly severed, apparently by the segmental fragment bone spike (Fig. 2). The extensor digitorum longus was found to be intact.

**Fig. 3:**
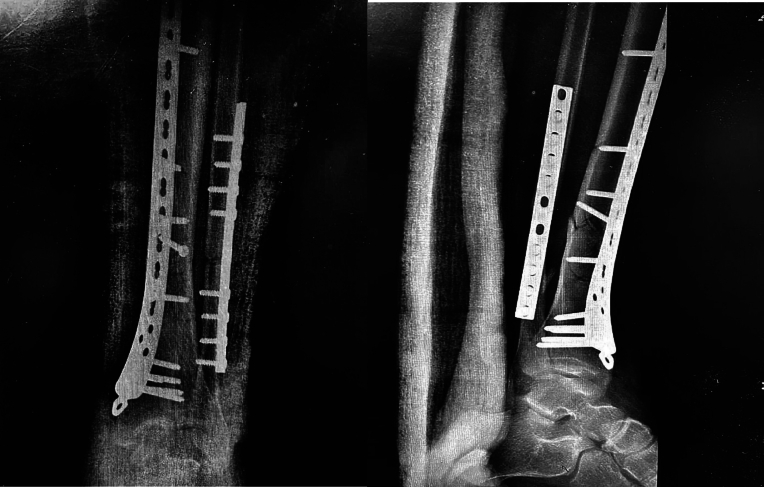
Postoperative anteroposterior and lateral radiographic imaging showing fracture at the junction of the middle and distal third of his tibia.

**Fig. 4:**
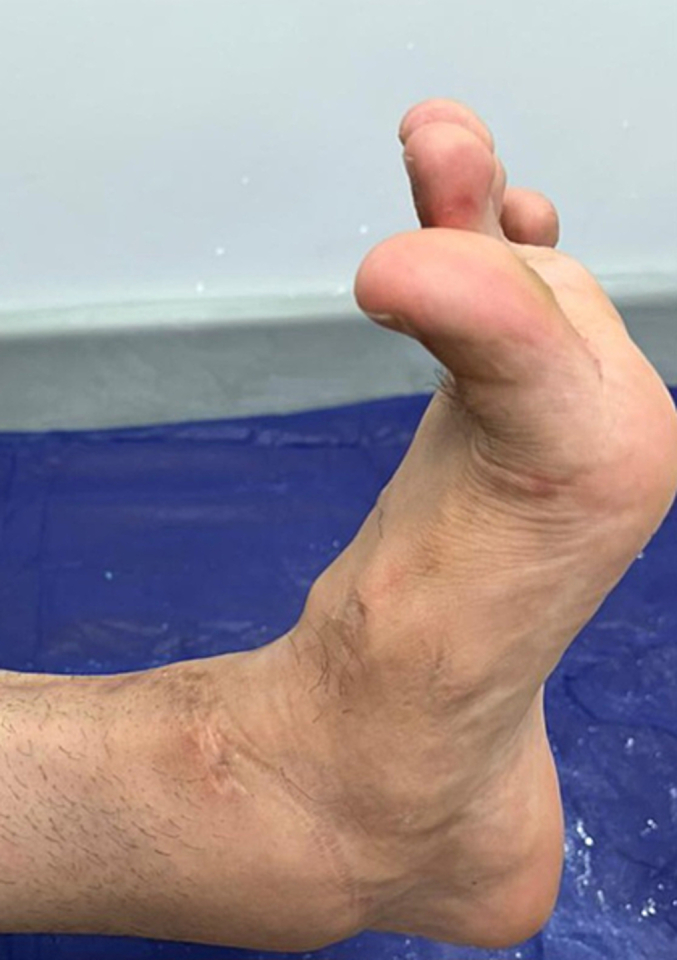
A clinical image that shows full dorsiflexion of the ankle and first toe has been obtained by 3 months postoperatively.

The fracture was reduced and plated with a limited-contact dynamic compression plate (LC-DCP; Synthes, Paoli, PA) titanium plate and 9 screws, and the tendons were subsequently sutured end-to-end with a modified Kessler stitch and reinforced by interrupted epitendinous polypropylene sutures. The tendon sheath was repaired with interrupted absorbable sutures; the fascia was closed over a drain and the skin was closed by using a Donati-Allgower stitch with its subcutaneous component on the medial side. The leg and ankle were put in a below-knee cast. Postoperative X-rays confirmed the fixation (Fig. 3). Intravenous antibiotics were administered for 48 h and low-molecular-weight heparin was administered for 6 weeks postoperatively for thromboprophylaxis. The leg was immobilized in a below-knee posterior splint, with the ankle joint maintained at 90°, for 6 weeks. The patient began partial weight-bearing and physiotherapy after the removal of the posterior splint. By 3 months postoperatively, he had achieved full active dorsiflexion of his right ankle and first toe (Fig. 4). On the follow-up after 2 years postoperatively, the Foot and Ankle Ability Measure (FAAM) activities of daily living (ADL) score was 93 % and the sports score was 81 %. On the final follow-up evaluation 3 years postoperatively, the patient showed full active dorsiflexion and plantarflexion of his ankle and first toe, very good power, and walked with a normal gait. At that time, the hardware was removed due to the pain, and the tibialis anterior and extensor hallucis longus tendons were completely healed with full restoration of their substance.

## 3 Discussion

Acute closed ruptures of the tibialis anterior and extensor hallucis longus tendon are infrequent entity [[3], [4], [5], [6], [7], [8], [9], [10]]. Brüning was the first who described a closed rupture of the tibialis anterior tendon in 1905 [4]. Tibialis anterior and extensor hallucis longus tendon ruptures can occur spontaneously without or with minimal trauma or as a result of penetrating or blunt trauma. The first category consists of the vast majority of tibialis anterior and extensor hallucis longus tendon ruptures that occur through the mid-substance of the tendon after minor trauma based on chronic inflammatory changes or fibrosis in pathologic conditions, such as gout, psoriasis, rheumatoid arthritis, myositis, systemic lupus erythematosus, hyperparathyroidism, hypothyroidism, diabetes or chronic steroid use [[1], [2], [3],[5], [6], [7], [8], [9], [10],^14^,^15^]. In such cases rupture usually occurs in the mid-substance of the tendon and a hypovascular zone has been proposed as a possible cause for this rupture. The most frequent site of spontaneous ruptures of the tibialis anterior tendon is an avascular zone 45 to 67 mm in length in the anterior half of the tibialis anterior tendon [16].

Traumatic tibialis anterior and extensor hallucis longus tendon ruptures in combination or as a result of bony fracture is extremely rare in closed trauma and only limited reports can be found in the literature. They are usually the result of direct penetrating or blunt trauma. Direct laceration of the tendon by the tibial fracture was reported in the rest 3 papers [[8], [9], [10], [11], [12],^18^]. In two of the tibialis anterior tendon rupture cases, the fractures were treated initially with a long-leg plaster cast, whereas the other was treated with an interlocking tibial nail but only one was depicted at the time of skeletal stabilization and therefore primarily repaired [18]. Of the two patients treated with a cast, one underwent a tendon reconstruction 11 months after injury and required aggressive physical therapy to correct the dorsiflexion deficit. The other patient was left with a residual equinus deformity.

In the present case reported above, the patient's tibia was broken. We believe that the segmental bone of the tibia that prolonged pressure contacts on the tibialis anterior and extensor hallucis longus tendon against the fracture edges caused the tendons to lacerate and rupture over a while, whilst the patient was waiting to be rescued. In this fracture pattern, the tibialis anterior and extensor hallucis longus tendon can be lacerated or entrapped between the two fragments, which may lead to fracture nonunion or disruption of the tendon, resulting in a residual equinus deformity if left untreated [13]. This is correlated with the findings at surgery, including the level of the rupture. Primary repair should always be the target whereas conservative management should be reserved for those unfit for surgery or elderly and inactive population.

In our case the tendon laceration could not be diagnosed preoperatively due to local swelling and pain therefore it was only discovered intraoperatively through the approach of tibia fixation. The absence of active dorsiflexion and a palpable tendon gap in the substance of the tibialis anterior tendon and extensor hallucis longus tendon should arouse suspicion regarding this rare injury. However, it is hard to predict if this injury could have been detected at that time of operation in case a different approach was used or in a different type of fracture. Therefore, it is advised that the examination of the tibialis anterior and extensor hallucis longus tendon is incorporated into the routine examination of a tibia fracture.

We believe that this is the first reported case in which the tendon laceration was diagnosed and treated immediately along with the tibial fracture, allowing full functional restoration of power to the tibialis anterior and extensor hallucis longus tendon and normal gait. This case as well as all prior reported tibialis anterior tendon ruptured cases were present in patients with tibia fracture. Thus, the initial ankle and toes dorsiflexion examination was disturbed and could have alerted the experienced surgeon of a potential tendon rupture.

## 4 Conclusion

Rupture of the tibialis anterior and extensor hallucis longus tendon complicating a closed tibial fracture is rare but should always be suspected in cases of oblique tibial fractures, especially when the fracture line is parallel to the line of pull of the tibialis anterior tendon. In conclusion, the persistent distribution of ankle and toes dorsiflexion examination and evaluation for a palpable gap in the soft tissues of the anterior aspect of the lower third of the patient's leg after tibia fracture requires further investigation. Interposing, tibialis anterior and other extensors between segments of tibia fracture can be found. Special attention must be exerted on tibia fracture.

## Consent

Written informed consent was obtained from the patient for publication and any accompanying images. A copy of the written consent is available for review by the Editor-in-Chief of this journal on request.

## Methods

The work has been reported in line with the SCARE criteria [19].

The work has been reported in line with the PROCESS criteria [20].

## Ethical approval

No ethics approval was needed as the case was encountered incidentally and it doesn't involve any human or animal experiment.

## Funding

This report did not receive any form of funding from any funding agencies in the public, commercial, or not-for-profit sectors.

## Author contribution

Amin Karami (Corresponding author), Saber Barazandeh Rad: study concept, writing the paper, review and editing.

Mohammad Pourmahmoudian: data collection

Mehrdad Sadighi, Meisam Jafari Kafiabadi, Adel Ebrahimpour: reviewing and validating the manuscript's credibility.

## Guarantor

Saber Barazandeh Rad.

## Conflict of interest statement

The authors have declared that no conflicts of interest exist. This research did not receive any specific grant from funding agencies in the public, commercial, or not-for-profit sectors.
